# Purification and Initial Characterization of 3-Hydroxybenzoate 6-Hydroxylase From a Halophilic *Martelella* Strain AD-3

**DOI:** 10.3389/fmicb.2018.01335

**Published:** 2018-07-06

**Authors:** Xin Chen, Hongzhi Tang, Yongdi Liu, Ping Xu, Yong Xue, Kuangfei Lin, Changzheng Cui

**Affiliations:** ^1^State Environmental Protection Key Laboratory of Environmental Risk Assessment and Control on Chemical Process, School of Resources and Environmental Engineering, East China University of Science and Technology, Shanghai, China; ^2^State Key Laboratory of Microbial Metabolism and School of Life Sciences and Biotechnology, Shanghai Jiao Tong University, Shanghai, China; ^3^Eco-environmental Protection Research Institute, Shanghai Academy of Agricultural Sciences, Shanghai, China

**Keywords:** moderate halophilic bacteria, 3-hydroxybenzoate, 3-hydroxybenzoate 6-hydroxylase, purification, FAD binding

## Abstract

3-Hydroxybenzoate 6-hydroxylase, an NADH-dependent flavoprotein, can convert 3-hydroxybenzoate which is an important intermediate in the biodegradation of many aromatic hydrocarbons. 3-Hydroxybenzoate is metabolized by entering the TCA cycle through the gentisate pathway. We found a putative 3HB6H gene from a cluster that potentially encodes for gentisic acid degradation from a halophilic *Martelella* sp. strain AD-3. The corresponding protein was expressed with an *N*-terminal His-tag and purified by Ni^2+^-nitrilotriacetic acid affinity chromatography. The protein showed an overexpressed band of about 46 kDa by SDS–PAGE, and it was also proven that the enzyme contains FAD by absorption spectroscopy and HPLC analysis. The optimal activity of 3HB6H from strain AD-3 was observed in phosphate buffer (pH 8.0) at 37°C without salinity (NaCl) and metal salts. The *K_m_* values of 3-hydroxybenzoate 6-hydroxylase were determined to be 72.6 ± 10.1 μM and 104.1 ± 18.2 μM for 400 μM NADH and 3-hydroxybenzoate, respectively. Site-directed mutagenesis showed that residues 305, 306 and 308 are important for FAD binding. In addition, we found that Tyr221 and Gln305 of 3HB6H from strain AD-3 are involved in substrate binding.

## Introduction

3-Hydroxybenzoate (3HB) is a compound commonly found in the environment, it is an intermediate of lignin biodegradation (Ko et al., 2009) and is used as a corrosion inhibitor (Narváez et al., 2005). It also appears in the gentisate pathway for the degradation of 2,5-xylenol, 3,5-xylenol and *m*-cresol (Hopper and Chapman, 1971; Hopper et al., 1971). There are two pathways for the aerobic degradation of 3HB, the gentisate (2,5-dihydroxybenzoate) pathway and the protocatechuate (3,4-dihydroxybenzoate) pathway through the catalysis of 3-hydroxybenzoate 6-hydroxylase (3HB6H) and 3-hydroxybenzoate 4-hydroxylase (3HB4H), respectively (Yano and Arima, 1958). 3HB6H is an NADH-dependent flavoprotein that contains FAD as a redox-active cofactor, and its quaternary structure is dimer (Montersino et al., 2013). This enzyme catalyzes the *para*-hydroxylation of 3-hydroxybenzoate to yield gentisate, in which consists of a reductive and oxidative half-reaction (Sucharitakul et al., 2013). In the catalytic reaction of 3HB6H, 3HB is preferred to bind as the first substrate, followed by NADH that leaves prior to the reaction with oxygen (Sucharitakul et al., 2013). Several 3HB6H have been purified and characterized, including 3HB6H from *Pseudomonas cepacia* (Wang et al., 1987), Ncgl2923 from *Corynebacterium glutamicum* ATCC 13032 (Yang et al., 2010), NagX from *Polaromonas naphthalenivorans* CJ2 (Park et al., 2007), 3HB6H from *Rhodococcus jostii* RHA1 (Montersino and van Berkel, 2012), and XlnD from *Pseudomonas alcaligenes* NCIMB 9867 (Gao et al., 2005). Among them, the crystal structure of 3HB6H from *Rhodococcus jostii* RHA1 has been reported that contains one molecule of FAD, one chloride-ion, and a phospholipid ligand (phosphatidylglycerol and phosphatidylethanolamine) in each subunit (Montersino et al., 2013). Recently, the new phospholipid ligand (phosphatidylinositol) was found in *Rj*3HB6H when the enzyme was homologously expressed in *Rhodococcus jostii* RHA1#2 (Montersino et al., 2017). In addition, site-directed replacements were able to affect the enzyme activity by affecting substrate binding, substrate hydroxylation or other properties (Liu et al., 2005; Montersino et al., 2013; Sucharitakul et al., 2015).

Halophilic *Martelella* sp. AD-3, isolated from highly saline petroleum-contaminated soil, was highly effective in degrading many PAHs with low-ring numbers, such as phenanthrene and anthracene, under broad salinities (0.1–15%) and varying pH values (6.0–10.0) (Feng et al., 2012; Cui et al., 2014). We found a gene (02545) encoding a putative 3HB6H (AZF01_02545) from the genomic sequence of halophilic *Martelella* sp. AD-3 (Cui et al., 2016). In strain AD-3, the gene is located upstream of *gdo*, whose expression and functions have been characterized previously (Huang et al., 2015). The 3HB6H from strain AD-3 has 57% identity with XlnD from *Pseudomonas alcaligenes* NCIMB 9867 as determined by BlastP search.

In this study, we report the expression of the gene (02545) encoding a putative 3HB6H in *Escherichia coli.* The corresponding enzyme was purified and characterized. In addition, we changed residues 221, 305, 306 and 308 by site-directed mutagenesis to study their possible involvement in substrate and FAD binding.

## Materials and Methods

### Chemicals and Media

3-Hydroxybenzoate (98% purity), salicylate (99% purity), 4-hydroxybenzoate (99% purity), gentisate (98% purity) and FAD (95% purity) were purchased from Aladdin (Shanghai, China), and NADH was purchased from Sangon Biotech (Shanghai, China). All other reagents and solvents used were analytical grade and the highest purity available. Luria-Bertani (LB) medium (10 g/L tryptone, 5 g/L yeast extract, and 10 g/L NaCl) was used for both culturing and cloning. Solid agar plates were prepared with the addition of 1.5% (w/v) agar to the LB liquid medium.

### Bacterial Strains, Plasmids, and Growth Conditions

*Escherichia coli* DH5α was used as the host for cloning experiments, while *E. coli* BL21(DE3) was used as the host for expressing the gene (02545). All the *E. coli* strains were grown in LB medium containing 50 mg/L kanamycin on a rotary incubator (200 rpm) at 37°C.

### DNA and Amino Acid Sequence Data Analysis

The amino acid sequences of the 3-hydroxybenzoate 6-hydroxylases, 3-hydroxybenzoate 4-hydroxylases and salicylate 1-hydroxylases from other strains were obtained from GenBank. All the homology searches were carried out on the NCBI BLAST server^1^ with nucleotide BLAST and protein BLAST. These obtained sequences were compared with the sequence from strain AD-3. Conserved binding domain searches were performed using Vector NTI DNA analytical software (version 11.0). Phylogenetic analysis was performed using MEGA 7. Protein structures were predicted by I-TASSER (Roy et al., 2010).

### Cloning and Overexpression of the Putative 3HB6H Gene

The gene (02545) was amplified from the genomic DNA of strain AD-3 using pfu DNA polymerase (Vazyme, China). The primers, which are the *Eco*RI (underlined) and *Hind*III (underlined) recognition sites, respectively, were designed as follows: 02545-forward (5′-CCGGAATTCATGTCAAACGTCGCAAATGA-3′) and 02545-reverse (5′-CCCAAGCTTTCAGGCCGAGACCGCGCCTT-3′). The amplified PCR product obtained was then excised with *Eco*RI and *Hind*III and ligated into pET28a(+), resulting in the recombinant plasmid designated pET28a-02545. The plasmid pET28a-02545 was subsequently transformed in *E. coli* BL21(DE3) to produce the protein with the His-tag located at *N*-terminal. *E. coli* BL21(DE3) carrying pET28a-02545 was cultured in LB medium containing 50 mg/L kanamycin and shaken at 37°C to an OD_600_ of 0.6–0.8. Then, after adding isopropyl β-D-thiogalactoside (IPTG) to a final concentration of 0.2 mM, the cultures were incubated for 16 h at 16°C or 30°C to express the protein. The cells were harvested by centrifugation at 9,000 × *g* for 20 min at 4°C and resuspended in binding buffer (25 mM Tris–HCl, 300 mM NaCl, and 20 mM imidazole, pH 8.0). The cells were sonicated 40 times for 3 s at 6 s intervals with an ultrasonic disintegrator in an ice bath and then centrifuged at 10,000 × g for 20 min at 4°C to separate the soluble protein from the insoluble membrane and protein aggregates. The supernatant was used for SDS–PAGE analysis. SDS–PAGE was performed using 12.5% acrylamide for the separating gel and 3.25% acrylamide for the stacking gel.

### Conversion of 3-Hydroxybenzoate by Using Resting Cells

Small-scale resting cell assays were performed as described previously (Panke et al., 1998) using an OD_600_ of 5.0 in 50 mM phosphate buffer (pH 8.0) containing 200 μM 3-hydroxybenzoate at 200 rpm and 30°C. During the course of the conversion, the concentrations of 3-hydroxybenzoate and gentisate were monitored by High Performance Liquid Chromatography (HPLC) and Liquid Chromatograph-Mass Spectrometer (LC-MS) systems (Agilent 1200 series, Agilent 1290 series ultra performance liquid chromatograph and TOFMS 6230, Agilent Technologies Inc., United States). For HPLC analysis, the sample of direct sampling was centrifuged at 10,000 × *g* for 2 min. And then the supernatant was filtered through a 0.22 μm millipore filter to prepare the samples. 10 μL of each sample was injected onto an Eclipse XDB-C18 column (4.6 × 150 mm; particle size, 5 μm) and run at 30°C with 50% methanol and 50% water (containing 1‰ formic acid) as the eluent at a flow rate of 0.8 mL/min. The concentrations of 3-hydroxybenzoate and gentisate were detected by UV spectroscopy at 235 nm and 320 nm. For LC-MS analysis, the conditions of liquid chromatography were the same as those of HPLC, and the mass spectrum adopted a negative ion mode.

### Purification of Putative 3HB6H From Strain AD-3

The cell cultures were induced for 16 h at 30°C and then disrupted by ultrasonic wave. After centrifugation, the supernatant was filtered through a 0.22 μm filter, and the resultant filtrate was applied to a 5 mL column of Ni-NTA agarose (GE, Healthcare, Little Chalfont, United Kingdom) that had been equilibrated with the binding buffer. After a wash with 30 mL of washing buffer (25 mM Tris–HCl, 300 mM NaCl, and 70 mM imidazole, pH 8.0), His_6_-tagged 3HB6H from strain AD-3 was eluted from the column using elution buffer (25 mM Tris–HCl, 300 mM NaCl, and 200 mM imidazole, pH 8.0). All the purification steps were carried out at 4°C.

### Protein Determination and Enzyme Assays

The concentration of the purified protein was measured by the Bradford method (Kruger, 1994). The Native-PAGE was performed using 7–15% gel. The protein was scanned using the full wavelength mode of a spectrophotometer after dilution with phosphate buffer (pH 8.0), and the wavelengths detected were from 250 to 550 nm. The identification of the protein binding to FAD was detected by HPLC. The protein was boiled for 3 min to isolate the cofactor, and the liquid chromatography conditions were the same as those used to determine the HspB cofactor (Tang et al., 2011). The concentration standard curve of FAD is shown in Supplementary Figure S4A. Lipid extraction was performed as described by van Berkel and coworkers (Montersino et al., 2013). Lipid identification was performed in solution containing 50% methanol and 50% acetonitrile (containing 2‰ formic acid), and was measured in a negative ion mode. 3HB6H activity was measured following NADH oxidation activity as a decrease in the absorbance at 360 nm (Montersino and van Berkel, 2012). Activity was assayed in 1 mL of reaction mixture containing 400 μM NADH, 400 μM 3-hydroxybenzoate and 10 μg enzyme extract in 50 mM phosphate buffer (pH 8.0) at 30°C with a UV-2550 spectrophotometer (Shimadzu, Kyoto, Japan). The molar absorption coefficient of NADH used to determine the reaction of 3-hydroxybenzoate was ε_360_ = 3.50 mM^-1^⋅cm^-1^ (Supplementary Figure S4B). Enzyme activity was assayed during 60 s after adding the enzyme to the reaction mixture. The hydroxylation efficiencies were determined by UV-2550 spectrophotometer and HPLC which detecting the change of NADH and 3HB, respectively.

### Kinetics Studies

A series of 3-hydroxybenzoate solutions ranging from 50 to 1000 μM was prepared to determine the *K_m_* values in 400 μM NADH. The *K_m_* values of 3HB6H for NADH were measured in 400 μM 3-hydroxybenzoate with concentrations of NADH ranging from 10 to 800 μM. Spectrophotometric assays (absorbance at 360 nm) were completed while maintaining a constant enzyme concentration in 50 mM potassium phosphate buffer (pH 8.0) at 30°C. Kinetic parameters were determined from Lineweaver-Burk plots.

### Site-Directed Mutagenesis

Site-directed mutagenesis was performed by using a recombinant PCR method. The primers are listed in Supplementary Table S1. The restriction sites were *Eco*RI (underline) and *Hind*III (underline). The recombinant pET28a that contained the mutant genes was transformed into *E. coli* BL21(DE3) followed by overexpression and purification. The strain growth, protein purification and enzyme assay methods are described above.

## Results

### Sequence Analysis of Putative 3HB6H From Strain AD-3

**FIGURE 1:**
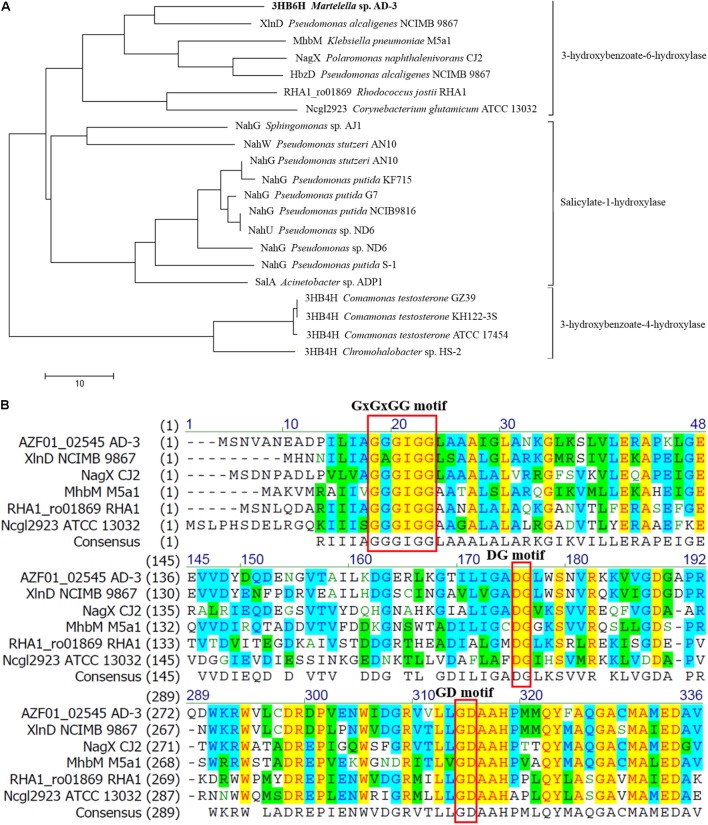
Amino acid sequence analysis results. **(A)** Neighbor-joining tree of 3-hydroxybenzoate 6-hydroxylase, salicylate hydroxylase and 3-hydroxybenzoate 4-hydroxylase. Aligned sequences are from halophilic *Martelella* sp. AD-3 (AMM83381.1), *Pseudomonas alcaligenes* NCIMB 9867 (Q9F131.1), *Klebsiella pneumoniae* M5a1 (AAW63416.1), *Polaromonas naphthalenivorans* CJ2 (Q3S4B7.1), *Corynebacterium glutamicum* ATCC 13032 (Q8NLB6.1), *Rhodococcus jostii* RHA1 (ABG93680.1), *Sphingomonas* sp. AJ1 (BAA19150.1), *Pseudomonas stutzeri* AN10 (AAD02157.1 and AAD02146.1), *Pseudomonas putida* KF715 (AAB35960.1), *Pseudomonas putida* G7 (AAA25897.1), *Pseudomonas putida* NCIB9816 (CAA58778.1), *Pseudomonas* sp. ND6 (AAP44222.1 and AAP44249.1), *Pseudomonas putida* S-1 (BAA61829.1), *Acinetobacter* sp. ADP1 (AAF04312.1), *Comamonas testosteroni* GZ39 (AAR25885.1), *Chromohalobacter* sp. HS-2 (AFK24467.1), *Comamonas testosteroni* ATCC 17454 (ABN58516.1), and *Comamonas testosteroni* KH122-3S (BAF34928.1). **(B)** Excerpts of sequences used for multiple sequence alignment performed by the Vector NTI program. Aligned sequences are from above.

Putative 3HB6H from strain AD-3 was found to belong to a family of group A flavoprotein monooxygenases that includes salicylate 1-hydroxylases, 3-hydroxybenzoate 4-hydroxylases and so on. (Montersino et al., 2011; Montersino and van Berkel, 2012; Huijbers et al., 2014). It was then compared with reported proteins and showed 34–57% identity with 3-hydroxybenzoate 6-hydroxylases, 23–28% identity with salicylate 1-hydroxylases, and only 15–17% identity with 3-hydroxybenzoate 4-hydroxylases. In particular, putative 3HB6H from strain AD-3 revealed 57% identity compared with XlnD from *Pseudomonas alcaligenes* NCIMB 9867 (Gao et al., 2005) and only 34% identity compared with *Rj*3HB6H from *Rhodococcus jostii* RHA1 (Montersino and van Berkel, 2012). A phylogenetic tree was constructed with 3-hydroxybenzoate 6-hydroxylases, salicylate 1-hydroxylases and 3-hydroxybenzoate 4-hydroxylases from other 17 strains and confirmed that it is most closely related to 3HB6H from *Pseudomonas alcaligenes* NCIMB 9867 (**Figure 1A**). The complete sequence alignment is shown in Supplementary Figure S1.

### Overexpression and Functional Verification of Putative 3HB6H From Strain AD-3 in BL21(DE3)

**FIGURE 2:**
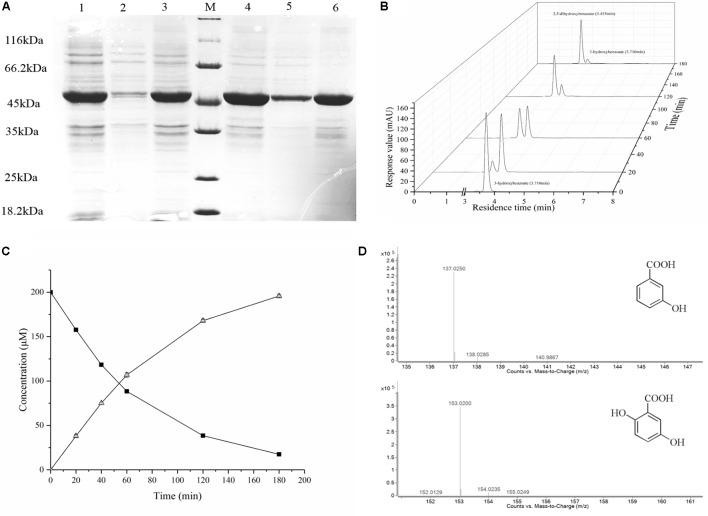
Overexpression and functional verification. **(A)** Lane M, protein molecular weight marker (MBI); Lane 1, cell culture at 16°C; Lane 2, the supernatant of the induced cells at 16°C; Lane 3, the precipitate of the induced cells at 16°C; Lane 4, cell culture at 30°C; Lane 5, the supernatant of the induced cells at 30°C; Lane 6, the precipitate of the induced cells at 30°C. **(B)** Enzymatic activity of the gene product from *E. coli*. Data show the conversion of 3-hydroxybenzoate into gentisate as determined by HPLC. The retention time of 3-hydroxybenzoate is 3.710 min, and the retention time of gentisate is 3.435 min. **(C)** The conversion of 3-hydroxybenzoate (top line, ▪) into gentisate (bottom line, Δ) as determined by HPLC. **(D)** The substrate and product identification by LC-MS.

The gene (02545) was cloned into the expression vector pET28a. *E. coli* BL21(DE3) containing the plasmid pET28-02545 was induced for 16 h in LB medium supplemented with 0.2 mM IPTG at 16°C and 30°C, respectively. The SDS–PAGE revealed an overexpressed band of about 46 kDa and showed the amount of soluble protein which induced at 30°C was more than that which induced at 16°C in same conditions (**Figure 2A**). To determine the protein encoded by the gene (02545) is a 3-hydroxybenzoate 6-hydroxylase, we used resting cell reaction to detect the conversion of hydroxybenzoate at 30°C by HPLC. The **Figures 2B,C** clearly showed that the concentration of 3-hydroxybenzoate decreased and the concentration of gentisate increased. The mass spectrometry data of LC-MS confirmed that the substracte was 3-hydroxybenzoate and the product was gentisate (**Figure 2D**). Thus, the protein encoded by the gene (02545) is a 3-hydroxybenzoate 6-hydroxylase.

### Purification and Characterization of 3HB6H From Strain AD-3

**FIGURE 3:**
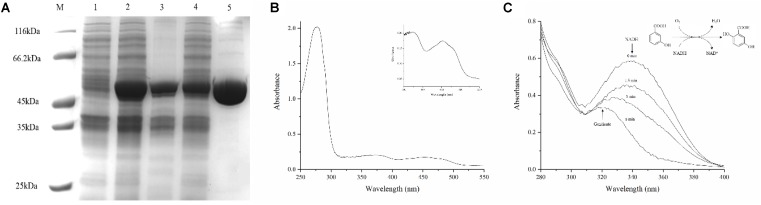
Purification of 3HB6H from strain AD-3. **(A)** Lane M, protein molecular weight marker (MBI); Lane 1, cell culture of cells transfected with the empty vector (pET28a) at 30°C; Lane 2, cell culture at 30°C; Lane 3, the precipitate of the induced cells at 30°C; Lane 4, the supernatant of the induced cells at 30°C; Lane 5, the purified 3HB6H protein. **(B)** The full-wavelength scanning results of 3HB6H. **(C)** The full-wavelength scanning *in vitro* conversion of 3-hydroxybenzoate by 3HB6H.

**FIGURE 4:**
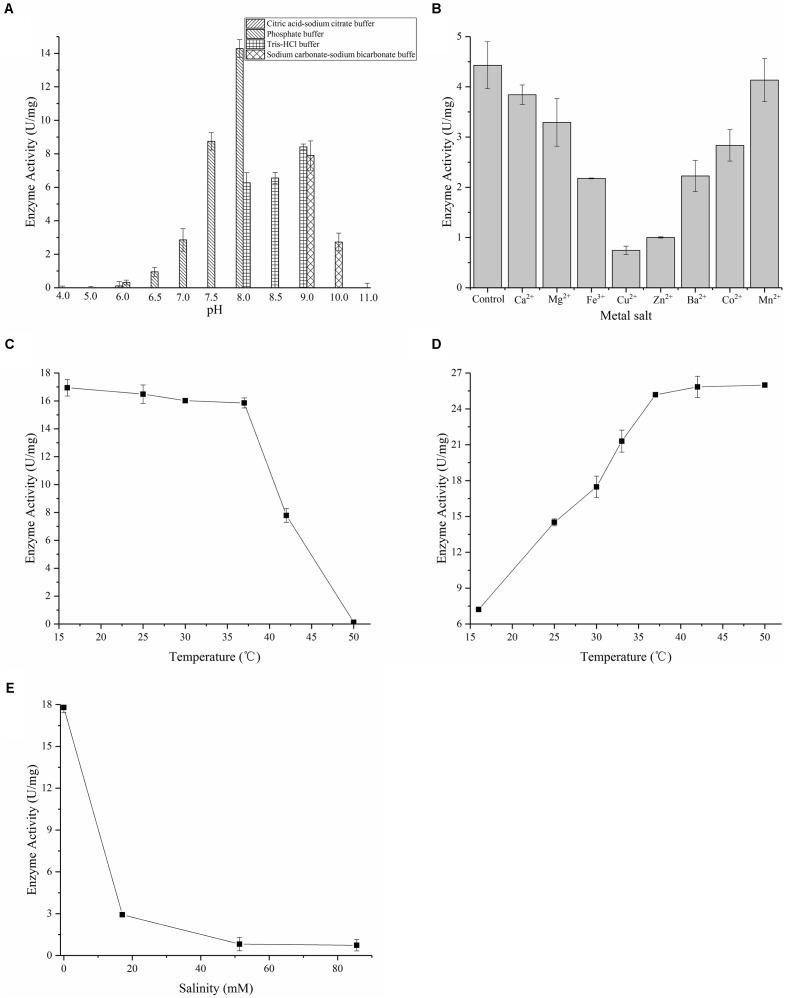
Characterization of 3HB6H from strain AD-3. **(A)** pH optimization of 3HB6H. 3HB6H has optimal activity in phosphate buffer (pH 8.0). **(B)** Effects of metal salts on enzyme activity; control without metal salt. Ca^2+^, Mg^2+^, Fe^2+^, Cu^2+^, Zn^2+^, Ba^2+^, Co^2+^ and Mn^2+^. **(C)** Temperature stability of 3HB6H activity. The enzyme activity was maintained at 16°C to 37°C. **(D)** Temperature sensitivity of 3HB6H activity; optimal activity was obtained at 37–50°C. **(E)** Salinity dependence of 3HB6H activity; maximum enzyme activity was observed without salt.

Protein 3HB6H was purified by using Ni-NTA, and the yellow protein was obtained. SDS–PAGE showed an overexpressed band of about 46 kDa, which is 3HB6H (**Figure 3A**). And the Native-PAGE showed a band of 90 kDa. Therefore, the 3HB6H from strain AD-3 might be dimeric protein (Supplementary Figure S2). The UV-VIS absorption spectrum showed maximum absorbance at 274, 375, and 452 nm, with a minimum at 410 nm and a shoulder at 480 nm (**Figure 3B**). The A274/A452 ratio of the FAD-saturated protein preparation was 11.5. The FAD concentration in the purified 3HB6H (0.29 mM) was 0.30 mM, and FAD/enzyme ratio was 1.02 ± 0.05 by HPLC analysis. The result of identifying protein-bound lipids showed that the 3HB6H expressed in *E. coil* contained phosphatidylglycerol, while phosphatidylethanolamine was not detected (Supplementary Figure S3 and Supplementary Table S2). In addition to its detection *in vivo*, the reaction resulting in the conversion of 3-hydroxybenzoate to gentisate was detected by ultraviolet spectrophotometry *in vitro*. The peak of NADH at 340 nm was reduced and moved to 320 nm (the peak of gentisate) over the course of time in reaction mixture containing 200 μM NADH and 200 μM 3-hydroxybenzoate (**Figure 3C**). The activity was measured 60 s after the addition of 3HB to reactions with different pH buffers. Citric acid-sodium citrate, phosphate, Tris–HCl, and sodium carbonate-sodium bicarbonate buffers were used for incubation at pH 4.0–6.0, 6.0–8.0, 8.0–9.0, and 9.0–11.0 with identical concentration (50 mM), respectively. 3HB6H showed maximal stability in phosphate buffer (pH 8.0). However, Tris–HCl (pH 8.0) obviously inhibited the activity of AZF01_02545 (**Figure 4A**). The effect of metal salts at 0.5 mM (Ca^2+^, Mg^2+^, Fe^3+^, Cu^2+^, Zn^2+^, Ba^2+^, Co^2+^, and Mn^2+^) was examined. Ca^2+^ and Mn^2+^ had no effect on enzyme activity. Mg^2+^, Fe^3+^, Cu^2+^, Zn^2+^, and Co^2+^ reduced enzyme activity. The addition of Cu^2+^ reduced enzyme activity to 16.8%, and even the addition of Zn^2+^ reduced enzyme activity to 22.6% (**Figure 4B**). To assess temperature stability, 3HB6H was incubated in phosphate buffer (50 mM, pH 8.0) for 60 min at temperatures ranging from 16 to 50°C for assay activity. The enzyme activity was maintained at 16–37°C, and quickly lost at higher temperatures. The enzyme precipitated initially at 42°C and precipitated completely at 50°C (**Figure 4C**). The influence of reaction temperature on enzyme activity was also investigated at the temperature range from 16 to 50°C in phosphate buffer (pH 8.0). The maximal enzyme activity was observed at 37°C, and the specific activity was 25.2 U/mg (**Figure 4D**).The effect of salinity on 3HB6H activity was measured in phosphate buffer (pH 8.0) at 30°C, with salinity ranging from 0 to 85.56 mM. With the increase in salinity, enzyme activity clearly decreased. The activity reached to 3% in 85.56 mM salinity (**Figure 4E**). Therefore, the maximum activity is 25.2 U/mg in optimal conditions which is phosphate buffer (pH 8.0) at 37°C without salinity (NaCl) and metal salts.

### Kinetic Properties

**FIGURE 5:**
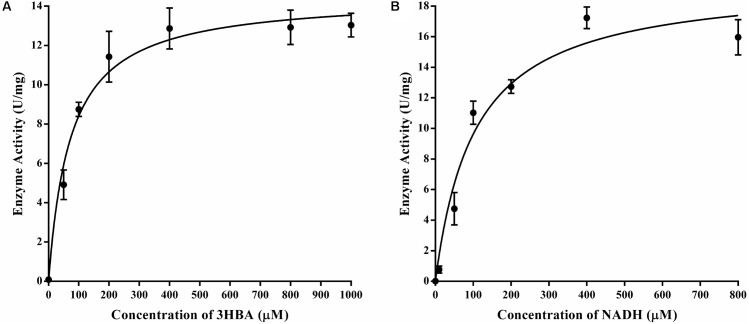
Enzyme kinetics of 3HB6H from strain AD-3. **(A)** A mixture containing 400 mM NADH in 50 mM phosphate buffer (pH 8.0) was mixed with an equal volume of enzyme (17.18 mg/ml). The *K_m_* values for 3-hydroxybenzoate **(B)** at a constant level of 3-hydroxybenzoate (0.4 mM) and various amounts of NADH; the *K_m_* values for NADH.

The purified 3HB6H displayed typical Michaelis-Menten kinetics, and Lineweaver-Burk plots of enzyme activity yielded an apparent *K_m_* of 72.6 ± 10.1 μM and *k_cat_* of 5.7 ± 0.2 s^-1^ for 3-hydroxybenzoate when the concentration of NADH was fixed at 400 μM and the concentrations of 3-hydroxybenzoate were varied (**Figure 5A**). At a constant level of 3-hydroxybenzoate (0.4 mM) and various amounts of NADH, the protein exhibited an apparent *K_m_* of 104.1 ± 18.2 μM and *k_cat_* of 7.8 ± 0.4 s^-1^ for NADH (**Figure 5B**).

### Site-Directed Mutations

Multiple sequence alignments with five enzymes known to be 3-hydroxybenzoate 6-hydroxylases (Montersino et al., 2017) with the Vector NTI program revealed three conserved motifs in the protein encoded by gene (02545), namely, the GxGxGG moiety, GD moiety and DG moiety (**Figure 1B**). The GxGxGG moiety (Gly15 Gly17 Gly19 Gly20 in 3HB6H from strain AD-3) is a conserved motif at the *N*-terminal region of NAD(P)H-dependent and FAD-containing oxidoreductases. This moiety is important for binding the ADP moiety of FAD (Wierenga et al., 1986). The GD motif (Gly297 Asp298 in 3HB6H from strain AD-3) is the second FAD-binding motif in flavoproteins and contacts the O3′ of the ribose moiety of FAD (Eggink et al., 1990). The DG motif (Asp166 Gly167 in 3HB6H from strain AD-3) serves a dual role in the recognition of FAD and NADH and is highly conserved among all flavoprotein hydroxylases studied (Eppink et al., 1997). Some sites in the GD region associated with FAD binding were obtained by I-TASSER.

We then obtained four mutants: Q305P, Y306H, A308G, and X (site mutations of 305, 306, and 308). In addition, we substituted a site (Tyr221) that was different from most salicylate hydroxylases in the same site (Phe) and obtained Y221F. The mutants were then purified; the enzyme activity and the amount of FAD binding of these mutant proteins compared to the wild-type protein are shown in **Table 1**. The enzyme activity of the mutant proteins was lower than the wild-type protein.

**Table 1:** The Kinetic Parameters of Wild Type and Mutant 3HB6H Enzymes.

Type	Activity (U/mg)	Amount of FAD binding (mol/mol)	Hydroxylation efficiency (%)	3HB			NADH		
				*K_m_* (μM)	*k_cat_* (s^-1^)	*k_cat_*/*K_m_*	*K_m_* (μM)	*k_cat_* (s^-1^)	*k_cat_*/*K_m_*
WT	15.4 ± 2.2	1.02 ± 0.05	88.0 ± 7.6	72.6 ± 10.1	5.7 ± 0.2	0.08	104.1 ± 18.2	7.8 ± 0.4	0.08
Q305P	3.3 ± 0.1	0.14 ± 0.02	66.8 ± 5.3	91.3 ± 13.5	1.6 ± 0.1	0.02	95.9 ± 23.2	1.6 ± 0.1	0.02
Y306H	3.4 ± 0.1	0.76 ± 0.10	82.4 ± 1.7	46.3 ± 6.9	1.5 ± 0.1	0.03	103.7 ± 13.6	1.6 ± 0.3	0.02
A308G	4.8 ± 0.3	0.34 ± 0.17	79.3 ± 1.0	38.0 ± 10.1	2.3 ± 0.1	0.06	33.7 ± 6.4	2.0 ± 0.1	0.06
X	1.2 ± 0.1	0.07 ± 0.01	68.1 ± 3.9	28.6 ± 7.3	0.5 ± 0.1	0.02	50.0 ± 9.7	0.6 ± 0.1	0.01
Y221F	2.6 ± 0.4	1.05 ± 0.1	47.2 ± 9.8	164.2 ± 14.0	1.7 ± 0.1	0.01	86.7 ± 10.8	1.0 ± 0.1	0.01

## Discussion

3HB6H plays an important role in the gentisate pathway of degradation 3HB in many bacteria. Previous reports were all found in non-halophilic organisms. This is the first report of a 3HB6H found in a halophilic *Martelella* strain.

### The Characteristics of 3HB6H From Strain AD-3

Amino acid sequence analysis revealed that 3HB6H from strain AD-3 has moieties similar to those of most 3-hydroxybenzoate 6-hydroxylases and salicylate hydroxylases; these moieties are the GxGxGG moiety, GD moiety and DG moiety (**Figure 1B**). Full-wavelength scanning showed a typical characteristic of flavoproteins (**Figure 3B**). The A274/A452 ratio of the FAD-saturated protein preparation was 11.5, and each 3HB6H subunit contained one FAD. This result showed that the 3HB6H from strain AD-3 is similar to *Rj*3HB6H (Montersino and van Berkel, 2012). 3HB6H from strain AD-3 also contained PGs like *Rj*3HB6H (Montersino et al., 2017), but we did not identify PEs. The PGs are the major lipid component of *Escherichia coli* cell membranes (Oursel et al., 2007). The phospholipids in 3HB6H are used to increase their dimerization strength (Montersino et al., 2017). Similar to other hydroxylases, 3HB6H from strain AD-3 utilizes NADH as the electron donor to complete the catalytic reaction (Steennis et al., 1973; Van Berkel and Wj, 1991). This protein converted 3-hydroxybenzoate to gentisate while consuming NADH *in vivo* and *in vitro* (**Figures 2B**, **3C**) and had no enzyme activity when salicylate and 4-hydroxybenzoate were used as substrates, similar to other 3-hydroxybenzoate 6-hydroxylases (data not shown) (Gao et al., 2005; Yang et al., 2010; Montersino and van Berkel, 2012). The molecular weight of 3HB6H from strain AD-3 is similar to that of most 3HB6H enzymes, such as RHA1_ro01869 (*Rhodococcus jostii* RHA1), XlnD (*Pseudomonas alcaligenes* NCIMB 9867), and NagX (*Polaromonas naphthalenivorans*) (Gao et al., 2005; Park et al., 2007; Montersino and van Berkel, 2012).

### The Key Conditions of Enzyme Activity

#### Metal Ions and Temperature

Comparing the effect of metal salts with XlnD, Ncgl 2923, and MhbM, Fe^3+^, Cu^2+^, Zn^2+^, Co^2+^, and Ba^2+^ reduced the activity of all of the enzymes (Gao et al., 2005; Park et al., 2007; Yang et al., 2010). Ca^2+^ and Mn^2+^ also reduce the enzyme activity except for 3HB6H from strain AD-3. Additionally, 3HB6H from AD-3 showed high activity (25.2 U/mg) in phosphate buffer (pH 8.0) at 37°C. This enzyme has relatively high activity after incubating at 16–37°C for 1 h, but the activity is 7.21 U/mg when the system environment is at 16°C. And there were high reaction speed at 42°C to 50°C, but the enzyme lost activity rapidly. Therefore, 37°C is the optimal temperature which guarantee no loss of enzyme activity and ensure to keep high activity.

### Chloride Ion

In the experiment to determine the effect of pH, 3HB6H showed 2.3 times higher activity in phosphate buffer (pH 8.0) than in Tris–HCl (pH 8.0). To explore whether 3HB6H catalysed 3-hydroxybenzoate under high salinity conditions (NaCl), we conducted a salinity experiment. *Martelella* strain AD-3, which is a moderate halophilic bacterium, can degrade 3-hydroxybenzoic acid in high salinity environments in which NaCl is the main source of salt. However, the activity of the purified 3HB6H decreased significantly as the amount of salinity increased. This phenomenon is similar to that observed with other flavoproteins; Cl^-^ has a strong inhibitory effect on flavoprotein monooxygenases (Steennis et al., 1973; Van Berkel and Wj, 1991; Montersino et al., 2013). In *Rhodococcus jostii* RHA1, the model of 3HB6H monomer contains one chloride ion that was found to bind in front of the flavin ring and the NH backbone atoms. This binding site corresponds to the niche predicted to host the oxygen atoms of the flavin-hydroperoxide adduct formed upon reaction of the reduced flavin with oxygen. These findings indicate that negatively charged chlorine can interfere with the binding and/or reactivity of the enzymes with oxygen, NADH, or both (Montersino et al., 2013).

### Binding to FAD and Substrate

Based on the reported structure of 3HB6H in *Rhodococcus jostii* RHA1 (Montersino et al., 2013) and the prediction of I-TASSER, Q305, Y306 and A308 are the binding sites for FAD. In this study, we found that the activity of the mutant enzymes decreased (**Table 1**). The FAD-binding ability of Y221F was similar to that of the wild-type enzyme. The *K_m_* of Y221F for 3HB was the highest among wild-type and mutants, and showed 2.3 times that of the wild-type, however, its enzymatic activity was less than that of the wild-type. Therefore, it was concluded that Y221F may be associated with substrate binding. In *Rj*3HB6H, Tyr217 is the important residue for substrate binding, which results in a hydrogen-bond interaction with the carboxylate group of 3HB. When substituting Tyr217, there is a substantial decrease or complete abolishment of substrate binding (Sucharitakul et al., 2015). Among them, X was the three-point mutation type, with the lowest amount of enzymatic activity and FAD binding. This phenomenon illustrated that the three sites involved in FAD binding. Q305 might play the most important role among them because the activity and binding FAD of Q305P were lower than those of A308G and Y306H. The *K_m_* of Q305P to 3HB was greater than that of the wild-type, and the binding amount of FAD was smaller than that of the wild-type. Therefore, Q305 was related to the binding of the substrate and FAD. This result validates previous findings in *Rj*3HB6H. When this site was changed to Glu, the enzyme lost activity. This site had a primary role in catalysis, possibly facilitating deprotonation of the substrate phenol (Montersino and van Berkel, 2012). When we changed Ala (308) to Gly, the activity and amount of FAD binding decreased. The reason may be the change of hydrophobic amino acid to hydrophilic amino acid and made it weaken the ability to combine with FAD. It coincided with the crystal structure of *Rj*3HB6H in which the A304 was related to bind FAD (Montersino and van Berkel, 2012).

## Author Contributions

HT and CC conceived and designed the experiments. XC performed the experiments. KL and YX analyzed the data. PX and YL contributed reagents, materials, and analysis tools.

## Conflict of Interest Statement

The authors declare that the research was conducted in the absence of any commercial or financial relationships that could be construed as a potential conflict of interest.

## Footnotes

1 http://www.ncbi.nlm.nih.gov/BLAST
