# Supplementary material for: Purification and Initial Characterization of 3-Hydroxybenzoate 6-Hydroxylase From a Halophilic Martelella Strain AD-3
Source: Front Microbiol. 2018 Jul 6;9:1335. doi: 10.3389/fmicb.2018.01335 (PMC6043778; doi:10.3389/fmicb.2018.01335)
Supplement: Supplementary file 1 [file Data_Sheet_1.docx]

***Supplementary Material***

**Purification and initial characterization of 3-hydroxybenzoate 6-hydroxylase from a halophilic *Martelella* strain AD-3**

**Xin Chen1, Hongzhi Tang^2#^, Yongdi Liu1, Ping Xu2, Yong Xue3, Kuangfei Lin1, and Changzheng Cui1^#^**

**^#^ Correspondence:** Hongzhi Tang: [tanghongzhi@sjtu.edu.cn](mailto:tanghongzhi@sjtu.edu.cn); Changzheng Cui: [cuichangzheng@ecust.edu.cn](mailto:cuichangzheng@ecust.edu.cn)

1. **Supplementary Data**

The accession number of amino acid sequence from 3HB6H is AMM83381.1, and the accession number of genome from strain AD-3 is CP014275.1.

The amino acid sequence from 3HB6H is as followings.

MSNVANEADPILIAGGGIGGLAAAIGLANKGLKSLVLERAPKLGEIGAGIQLGPNAFHAFDYLGVGDAARAIAVYIDNLRLMDAIDGQEITRIPLDDAFRARFRNPYAVIHRGDLHGVFLKACEDHPLVSLRTNAEVVDYDQDENGVTAILKDGERLKGTILIGADGLWSNVRKKVVGDGAPRVSGHTTYRSVIPTEDMPEDLRWNAATLWAGPKCHIVHYPLRGWKLFNLVVTYHNHAPSPEAGVPVSHEEVRKGFEHVAPVARQIIERGQDWKRWVLCDRDPVENWIDGRVVLLGDAAHPMMQYFAQGACMAMEDAVAISHVLGEEGGPLDEALARYQELRALRTARVQLQSREIGQHVYHPGGAHAALRNAVMRSKSPDDWYDIVSWLYGSTGLEGAVSA

The gene sequence is also as followings.

ATGTCAAACGTCGCAAATGAAGCAGACCCGATCCTGATCGCCGGCGGCGGCATCGGCGGCCTTGCCGCCGCCATCGGTCTGGCGAACAAGGGGCTGAAGTCGCTGGTGCTGGAGCGCGCGCCGAAGCTCGGCGAAATCGGCGCCGGCATTCAGCTCGGCCCAAACGCCTTTCATGCCTTCGATTATCTGGGCGTCGGCGATGCCGCGCGGGCGATTGCCGTCTATATCGACAATCTACGCCTGATGGATGCCATTGACGGTCAGGAGATCACCCGCATTCCGCTGGACGATGCCTTCCGCGCCCGCTTCAGAAACCCTTATGCCGTGATCCATCGCGGCGACCTCCACGGCGTGTTCCTGAAAGCCTGCGAGGATCATCCTCTGGTCAGCCTGCGCACCAATGCCGAAGTGGTCGACTACGATCAGGACGAAAACGGCGTCACCGCGATCCTGAAGGACGGAGAACGGCTGAAGGGGACAATCCTGATCGGCGCAGACGGGCTGTGGTCCAATGTCCGCAAGAAGGTGGTCGGCGACGGCGCGCCGCGTGTTTCCGGCCACACCACCTATCGTTCTGTCATCCCGACGGAAGACATGCCGGAAGACCTGCGCTGGAACGCCGCAACGCTCTGGGCCGGGCCGAAATGTCACATCGTCCATTATCCGCTCAGGGGCTGGAAACTCTTCAACCTGGTGGTGACCTATCACAACCACGCGCCCTCGCCGGAAGCCGGCGTGCCGGTTTCCCATGAGGAGGTCCGCAAGGGTTTCGAACACGTGGCGCCGGTCGCGCGCCAGATCATCGAGCGCGGTCAGGACTGGAAGCGATGGGTTCTCTGCGACCGCGACCCGGTGGAAAACTGGATCGACGGTCGCGTCGTCCTGCTGGGCGACGCCGCCCATCCGATGATGCAGTATTTCGCCCAGGGCGCTTGCATGGCCATGGAGGATGCCGTGGCCATTTCCCATGTGCTCGGCGAGGAGGGCGGTCCGCTTGACGAGGCCCTTGCCCGCTACCAGGAACTGCGCGCGCTGCGCACTGCGCGCGTTCAGCTGCAGTCGCGTGAAATCGGCCAGCACGTCTATCACCCCGGCGGCGCGCACGCCGCGCTTCGCAACGCGGTGATGCGTTCCAAGTCGCCGGATGACTGGTACGATATCGTCTCCTGGCTCTACGGCTCCACCGGCCTTGAAGGCGCGGTCTCGGCCTGA

1. **Supplementary Figures and Tables**
   1.
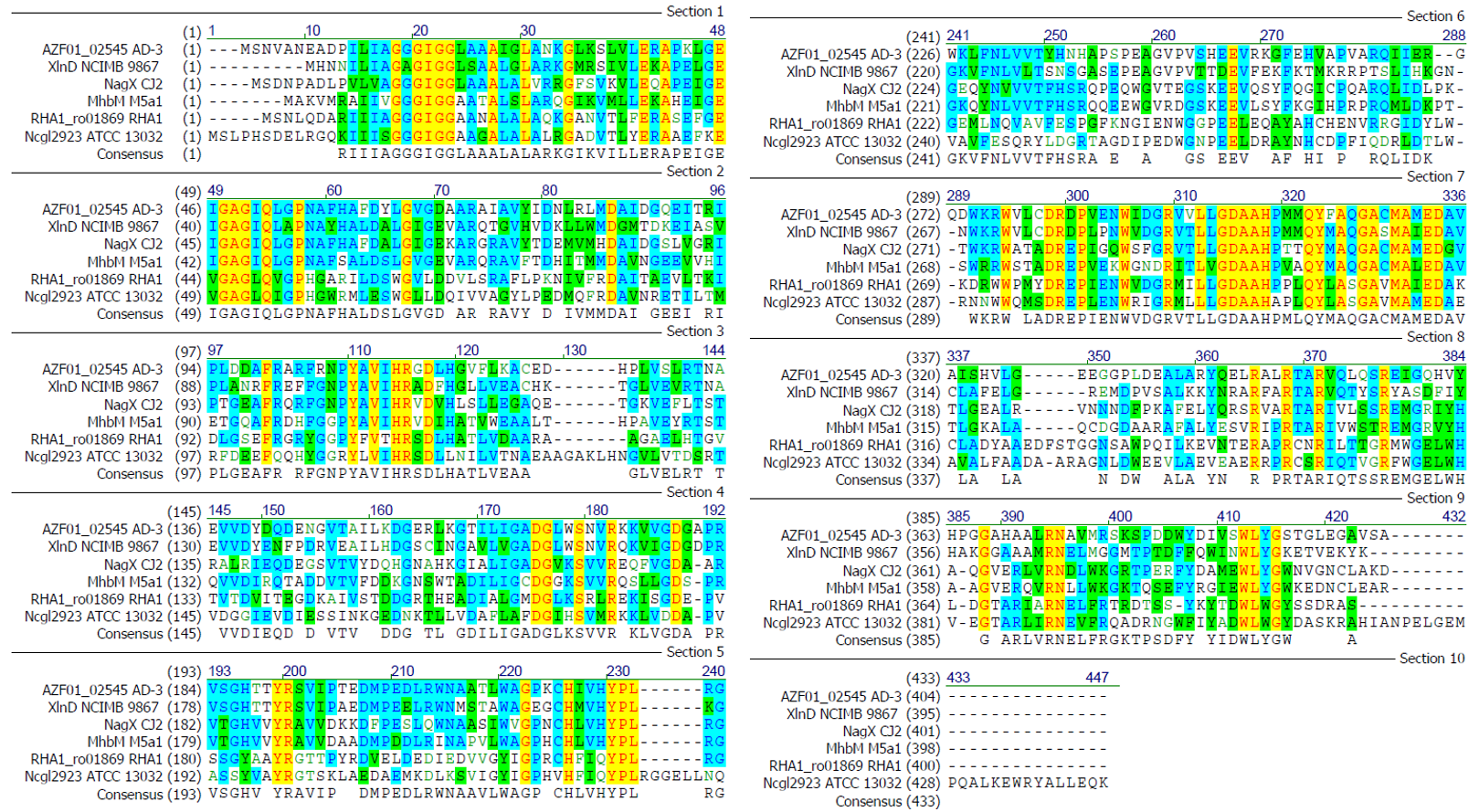
**Supplementary Figures**

**Fig. S1. Amino acid sequence analysis results.** All sequences used for multiple sequence alignment performed by the Vector NTI program. Aligned sequences are from above. Aligned sequences are from halophilic *Martelella* sp. AD-3 (AMM83381.1), *Pseudomonas alcaligenes* NCIMB 9867 (Q9F131.1), *Klebsiella pneumoniae* M5a1 (AAW63416.1), *Polaromonas naphthalenivorans* CJ2 (Q3S4B7.1), *Corynebacterium glutamicum* ATCC 13032 (Q8NLB6.1), *Rhodococcus jostii* RHA1 (ABG93680.1).


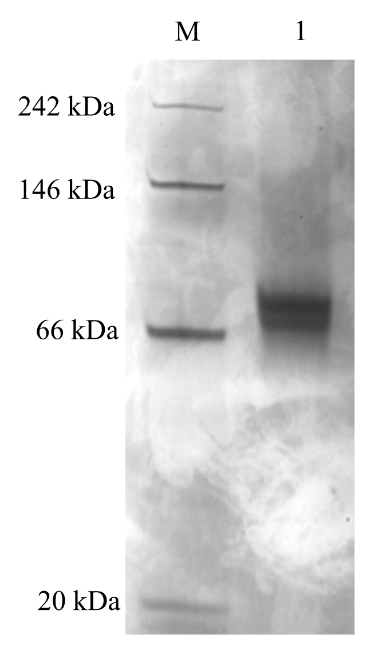


**Fig. S2. Native-PAGE of purified 3HB6H from strain AD-3.** Lane M, protein molecular weight marker (MBI); Lane 1, 2.5 μg 3HB6H

**
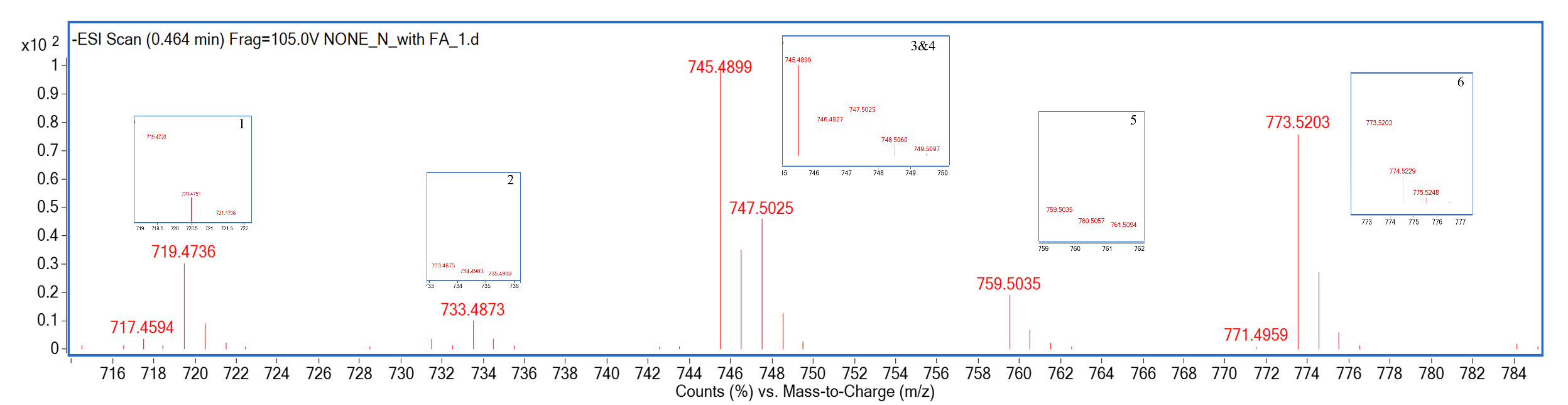
**

**Fig. S**3**. The Lipids identification by mass spectrometry.** 3HB6H from strain AD-3 also contained PGs (PG C14:0/C18:1; PG C15:0/cyC17:0; PG C16:0/C16:1; PG C16:0/cyC17:0; PG C18:1/C16:1; PG C16:0/C18:1; PG C18:1/cyC17:0; PG C18:1/C18:1)

**
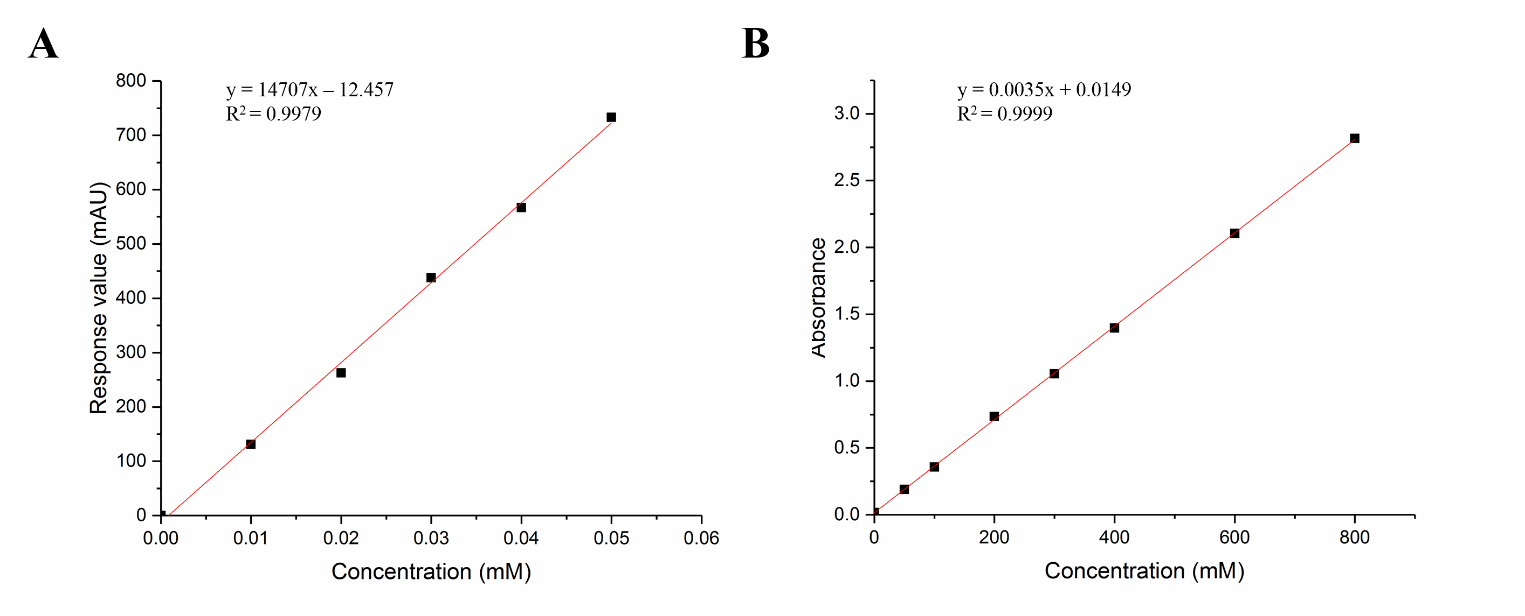
**

**Fig. S4. The standard curve of FAD and NADH.** (A) the standard curve of FAD; (B) the standard curve of NADH

**1.2 Supplementary Tables**

Site-directed mutagenesis was performed by using a recombinant PCR method. The primers are listed in Table S1**.**

**Table S1** Primers for site-directed mutagenesis

| Points | F’ | R’ |
| --- | --- | --- |
| Q305P | 5’-CATCCGATGATGCCGTATTTCGCCCAG-3’ | 5’-CTGGGCGAAATACGGCATCATCGGATG-3’ |
| Y306H | 5’-CCGATGATGCAGCATTTCGCCCAGG-3’ | 5’-CCTGGGCGAAATGCTGCATCATCGG-3’ |
| A308G | 5’-GATGATGCAGTATTTCGGCCAGGGCGCTTGCATG-3’ | 5’-CATGCAAGCGCCCTGGCCGAAATACTGCATCATC-3’ |
| X | 5’-CATCCGATGATGCCGCATTTCGGCCAGGGCGCTTGCATG-3’ | 5’-CATGCAAGCGCCCTGGCCGAAATGCGGCATCATCGGATG-3’ |
| Y221F | 5’-GAAATGTCACATCGTCCATTTCCCGCTCAGGGGCTGGAAAC-3’ | 5’-GTTTCCAGCCCCTGAGCGGGAAATGGACGATGTGACATTTC-3’ |

The result of identifying protein-bound lipids followed as Table S2. It coincided with Fig S3.

**Table S2.** Lipid identification by mass spectrometry

PG, phospatidylglycerol; cy, cyclic.

| number | [M-H]^-^ | PLs |
| --- | --- | --- |
| 1 | 719 | PG C14:0/C18:1 |
|  |  | PG C15:0/cyC17:0 |
|  |  | PG C16:0/C16:1 |
| 2 | 733 | PG C16:0/cyC17:0 |
| 3 | 745 | PG C18:1/C16:1 |
| 4 | 747 | PG C16:0/C18:1 |
| 5 | 759 | PG C18:1/cyC17:0 |
| 6 | 773 | PG C18:1/C18:1 |
